# Development of an Evidence-Based Conceptual Model of the Health Care Sector Under Digital Transformation: Integrative Review

**DOI:** 10.2196/41512

**Published:** 2023-06-08

**Authors:** Jens Konopik, Dominik Blunck

**Affiliations:** 1 Institute of Management Friedrich-Alexander-Universität Erlangen-Nürnberg Nuremberg Germany

**Keywords:** digital transformation, health care, Healthcare 4.0, digital health, eHealth, conceptual model, literature review, grounded theory, integrative review

## Abstract

**Background:**

Digital transformation is currently one of the most influential developments. It is fundamentally changing consumers’ expectations and behaviors, challenging traditional firms, and disrupting numerous markets. Recent discussions in the health care sector tend to assess the influence of technological implications but neglect other factors needed for a holistic view on the digital transformation. This calls for a reevaluation of the current state of digital transformation in health care. Consequently, there is a need for a holistic view on the complex interdependencies of digital transformation in the health care sector.

**Objective:**

This study aimed to examine the effects of digital transformation on the health care sector. This is accomplished by providing a conceptual model of the health care sector under digital transformation.

**Methods:**

First, the most essential stakeholders in the health care sector were identified by a scoping review and grounded theory approach. Second, the effects on these stakeholders were assessed. PubMed, Web of Science, and Dimensions were searched for relevant studies. On the basis of an integrative review and grounded theory methodology, the relevant academic literature was systematized and quantitatively and qualitatively analyzed to evaluate the impact on the value creation of, and the relationships among, the stakeholders. Third, the findings were synthesized into a conceptual model of the health care sector under digital transformation.

**Results:**

A total of 2505 records were identified from the database search; of these, 140 (5.59%) were included and analyzed. The results revealed that providers of medical treatments, patients, governing institutions, and payers are the most essential stakeholders in the health care sector. As for the individual stakeholders, patients are experiencing a technology-enabled growth of influence in the sector. Providers are becoming increasingly dependent on intermediaries for essential parts of the value creation and patient interaction. Payers are expected to try to increase their influence on intermediaries to exploit the enormous amounts of data while seeing their business models be challenged by emerging technologies. Governing institutions regulating the health care sector are increasingly facing challenges from new entrants in the sector. Intermediaries increasingly interconnect all these stakeholders, which in turn drives new ways of value creation. These collaborative efforts have led to the establishment of a virtually integrated health care ecosystem.

**Conclusions:**

The conceptual model provides a novel and evidence-based perspective on the interrelations among actors in the health care sector, indicating that individual stakeholders need to recognize their role in the system. The model can be the basis of further evaluations of strategic actions of actors and their effects on other actors or the health care ecosystem itself.

## Introduction

### Overview

Digital transformation has gained an important role in strategic management research [1]. Vial [2] describes digital transformation in the most widely accepted definition as “a process that aims to improve an entity by triggering significant changes to its properties through combinations of information, computing, communication, and connectivity technologies.” Digital transformation improves an entity and redefines its value proposition for its stakeholders [3], thus having implications for the whole structure of the entity [4].

Due to the widespread implications of digital transformation, it is of high priority among the vast majority of organizations [5]. This also includes the health care sector as one of the most critical economic, social, and political part of societies [6]. Digital transformation is expected to be the key driver of clinical outcomes [7] and is fundamental to cost-saving initiatives [8]. It offers great possibilities for stakeholders in the health care sector to fundamentally change the provision of services [9]. Despite the constant introduction of new technologies, this disruptive change is yet to materialize [10]. Although the health care sector has been ever evolving, to this date, this sector is evolving at a very slow pace [11-13]. This leads to the health care sector being considered a latecomer industry when it comes to the introduction of digital transformation [14]. Although the substantial impact of specific digital technologies in health care has been widely discussed [15], the wide-ranging impact of digital transformation has hardly been discussed. Consequently, academic research focusing on a holistic perspective of digital transformation in the health care sector has been underrepresented [16].

### Characteristics of Digital Transformation in Health Care

Vial’s [2] widely accepted definition of digital transformation highlights the substantial changes to an entity with the means of (digital) technologies. Other analyses focusing on definitions [3], models and frameworks [17], or characteristics and drivers of digital transformation [18] unanimously confirm the crucial role of digital technologies as the central means of disruption. Digital technologies refer to combinations of information, computing, communication, and connectivity technologies or technologies that are new for the respective entity and cause strategic alignment of business processes. This includes technologies that are commonly referred to as disruptive technologies [19]. Despite the highly regulated nature of the health care sector [20], it has recently witnessed a growth in the adoption of novel technologies [21]. Alluding to the intertwining of physical and software components and the incorporation of novel information and communications technologies into corporations, known as Industry 4.0, the academic literature refers to this development in the health care sector as Healthcare 4.0. It constitutes the technology-enabled shift to patient-centered organizational structures that enable real-time customization of health care for patients [22] based on a data-driven predictive medicine approach [23].

In this study, the term entity refers to “an individual organization, business network, industry, and society” [3] in the health care sector. This broad interpretation of the term highlights the wide-ranging impact of digital transformation and the imperative need to consider whole value networks instead of single organizations [24,25]. Fundamental changes in business and organizational activities, processes, competencies, and business models [26] are fueled by ecosystem possibilities. Digital transformation facilitates the cocreation of value among various stakeholders by the combination of complementarities and interdependencies between entities inside the network [27] to address large, complex problems [28]. This allows actors in the health care sector to shift their business models from simply providing reactive medical care toward prevention, chronic disease management, and the joint and holistic delivery of health [29].

The outcome of digital transformation is a strategic alignment that drives both capability-driven changes (eg, business model innovation and radical changes in offerings to customers) as well as efficiency and productivity changes (eg, cost reduction and error elimination) [2,3,30]. This results in major changes to the creation of value of the organization as well as to its stakeholders. These 2, however, are often interconnected, as network effects of digital and connected services are increasingly becoming the key differentiator and driver of value creation [30].

### Problem Statement, Aim, and Research Questions

The adoption of digital technologies in digital transformation is accompanied by the redefinition of the meaning of health and the provision of related services [31]. Contrary to the expectations of scholars, the digital transformation of the health care sector in general is underrepresented in academic discussions [16]. With most studies providing a narrow perspective on the application of novel technologies in the health care sector [32], only few perspectives focus on the holistic nature of digital transformation, encompassing society and organizations of the sector alike [31]. This void of research on unifying perspectives impedes discussions on fundamental shifts in the health care sector [33]. A unifying perspective is necessary to foster a common understanding of the changing nature of the sector and to facilitate the evaluation of practitioners and academic scholars of strategic actions inside the sector alike. This is particularly valuable for policy makers to guide regulatory measures as well as organizational entities in the sector to structure their strategic decision-making.

Recent publications have therefore called for further discussions on the new perceptions of health among various stakeholders [34] and the perception of value in the health care sector in general [35]. With the digital transformation being overdue in the health care sector and the ongoing mindset shift among stakeholders [11], this calls for a reevaluation of the current state of digital transformation in health care. To the best of our knowledge, however, no evidence-based study has assessed digital transformation in the health care sector from a holistic perspective.

Accordingly, this study answers two research questions: (1) What impact does digital transformation have on the value creation of the most essential stakeholders? and (2) What impact does digital transformation have on the relationships among the most essential stakeholders? To answer these questions, we derived evidence-based results from a systematic integrative review, supported by grounded theory. These results were then synthesized into a unifying model.

## Methods

### Overview

This section describes the 2-staged methodology used in this qualitative study. First, we identified the most relevant stakeholders in the health care sector using a scoping review approach. On the basis of these results, we followed the integrative review process by Whittemore and Knafl [36], which is shaped by a systematic literature search, data evaluation, and data analysis, to identify relevant articles to answer both research questions. To systematize the results in this emerging and growing stream of research, the principles of conceptual modeling theory were applied. These include the combination of various concepts, key factors, or constructs [37-40]; the explanation of the presumed relationships between these concepts or constructs [37,39,40]; and the provision of a soft interpretation [41] of the intentions of the involved entities via a graphical form [39,42].

### Stakeholders in the Health Care Sector

Although the literature provides some concepts of stakeholder classifications for the health care sector, their ability to generalize the stakeholder landscape in health care remains unclear. To create good prerequisites for the following integrative review and to ensure an adequate fit to the desired level of abstraction in our study, an appropriate stakeholder concept must be derived from the literature. To break the trade-off between efficiency and quality at this stage, a scoping review approach was chosen, as it is suggested for identifying key characteristics and factors related to a concept [43].

On the basis of this scoping review approach, a nonsystematic literature search was conducted. Search terms such as stakeholders and health care were used in Google and Google Scholar to identify relevant concepts. Extensive forward and backward searches further augmented the results. Following the World Health Organization’s definition of a health system, results must aim to provide a holistic representation of the health care sector and identify entities (eg, organizations, people, and institutions) [44] participating in it.

The results were then analyzed based on a grounded theory approach by Strauss and Corbin [45], with a generic level of abstraction needed for a holistic conceptualization of the health care sector. Stakeholders should be analyzed based on the core value they provide for the ecosystem itself or other stakeholders in the system. The initial coding process consisted of a word-by-word analysis of the identified stakeholder concepts. Identified stakeholders were coded in vivo, that is, in the exact form they appear in the stakeholder concepts [46]. In the next stage, axial coding, the identified stakeholders were grouped together into higher-order categories. For example, the stakeholders “hospitals,” “rehab center,” and “clinicians” were grouped into the higher-order category “medical treatments.” During this stage, the categorizations were constantly checked and regrouped until a sound categorization of the stakeholders was achieved, following the Mutually Exclusive and Collectively Exhaustive (MECE) principle. In the last stage, the selective coding stage, the final codes of the stakeholder categorization were identified. At this stage, data of little or no importance to the core categories and supporting properties were filtered [47]. This step involved repeated revisions of the results until the stakeholders in the health care sector were ultimately identified.

The scoping review yielded 12 relevant stakeholder conceptualizations [31,48-58]. The goal of the conceptual model, to focus on the most crucial elements of the health care sector, guided the subsequent coding process. This led to the identification of 96 relevant in vivo segments, which were subsequently grouped into higher-order concepts to derive relevant stakeholders. Notably, because of their broad scope or lack of clarity, a total of 15 segments could not be grouped with other stakeholders.

### Integrative Review

#### Search Strategy

Conducting the integrative review, we complied with the PRISMA (Preferred Reporting Items for Systematic Reviews and Meta-Analyses) extension for Scoping Reviews statement, and the completed checklist is provided in Multimedia Appendix 1 [59]. Before the actual literature search, a pilot search was conducted to assess the feasibility of the literature search and to identify relevant keywords. This step indicated that a large number of potentially relevant articles associated digital transformation in health care with terms such as “Health Care 4.0,” “Healthcare 4.0,” and “Health 4.0.” Furthermore, no relevant synonyms for the term “digital transformation” could be identified in Web of Science or PubMed articles. Test runs in databases showed that the identification of relevant articles would not benefit from adding specific technologies to the search terms, as these terms are predominantly accompanied by digital transformation–related and Healthcare 4.0–related constructs.

Accordingly, the search term (health* AND digital transformation) OR Health Care 4.0 OR Healthcare 4.0 OR Health 4.0 was used to combine the domains of health care and digital transformation.

The databases Web of Science, PubMed, and Dimensions were chosen to carry out the literature search. Web of Science and PubMed qualify as principal resources for literature searches and capture the involved disciplines [60]. Dimensions complements these 2 databases by providing a larger data set and additional nonindexed publications [61]. The search terms were generated in compliance with the specific syntax requirements of each of the databases (Multimedia Appendix 2).

**Figure 1:**
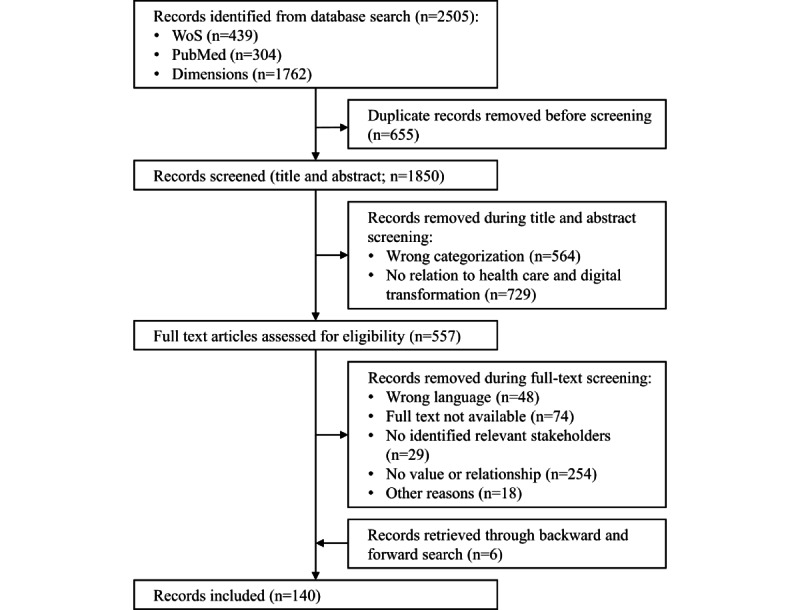
PRISMA (Preferred Reporting Items for Systematic Reviews and Meta-Analyses) flow diagram describing the literature search (adapted from the study by Page et al [63]). WoS: Web of Science.

The following inclusion and exclusion criteria were based on the research questions. First, the search results must address the spectrum of digital transformation in the context of the health care sector. Strategic changes to an entity must be triggered by digital technologies. As inductively derived from the relevant literature [2,3,30], and described in the *Introduction* section, these responses must either improve the efficiency and effectiveness of internal processes of an organization, satisfy needs inside the ecosystem better or at lower costs than existing options, or redefine either its value proposition for or the relationship with its stakeholders. A mere digitization of a process without further strategic implications would not be sufficient to meet this criterion. Second, the entities referred to in the first criterion must include at least 1 of the identified stakeholders. Furthermore, articles must be written in German or English (Table 1). Regarding the date of publication, we decided not to further limit the results. Although the literature indicates that the vast majority of high-quality publications on digital transformation emerged after 2010 [62], a pilot search revealed that only a fraction of the search results is expected to be published before 2010, allowing the inclusion of these results with reasonable effort. Two independent researchers screened and evaluated the identified records. Disagreements were discussed and resolved by consensus (Figure 1 depicts the PRISMA flow diagram [63]).

**Table 1:** Inclusion and exclusion criteria.

Criteria	Inclusion	Exclusion
Scope of digital transformation	Strategic change to entity triggered by digital technologies	No strategic change
Outcome of strategic change	Improved efficiency or effectiveness of internal processes [2,3], improved satisfaction of needs inside the ecosystem or satisfied at lower cost [3,30], and redefinition of value proposition or relationship [2,3,30]	Unspecified or other outcomes
Considered entities	Identified stakeholders	Other entities than stakeholders
Language	English or German	Not English or German

The systematic database search for the integrative review process yielded 2505 records, of which 140 (5.59%) were considered relevant (Figure 1).

#### Data Evaluation

Although the methodology by Whittemore and Knafl [36] suggests a data evaluation stage, no traditional quality assessment of the included search results was performed. This is because the pilot search revealed that insights into the impact of digital transformation on relevant stakeholders, as the unit of analysis of this study, are often only a byproduct or one of many elements of interest of the relevant literature. This phenomenon has also been observed by Paré et al [64], who proposed to consider the relevance (thematic fit of the study) and rigor as the 2 dimensions to judge the results. Consequently, to incorporate inputs straddling various research streams and originating from a heterogeneous range of study designs, rather than limiting the results to studies meeting specific methodological standards [65,66], no conventional quality appraisal methods from other aggregative reviews, such as quality checklists, were used. Instead, the results were required to meet the inclusion criteria mentioned in the *Search Strategy* subsection in the *Methods* section.

#### Data Analysis

During the data analysis stage, grounded theory [45] was used to systematically structure the insights from the identified relevant literature. According to a comparison of theory-building methods [67], grounded theory is particularly valuable for generating new insights through the continuous interplay between analysis and data collection. It is an inductive-driven qualitative research method used for the conceptual development phase of the theory-building process [67], which makes it an appropriate method for this study.

**Figure 2:**
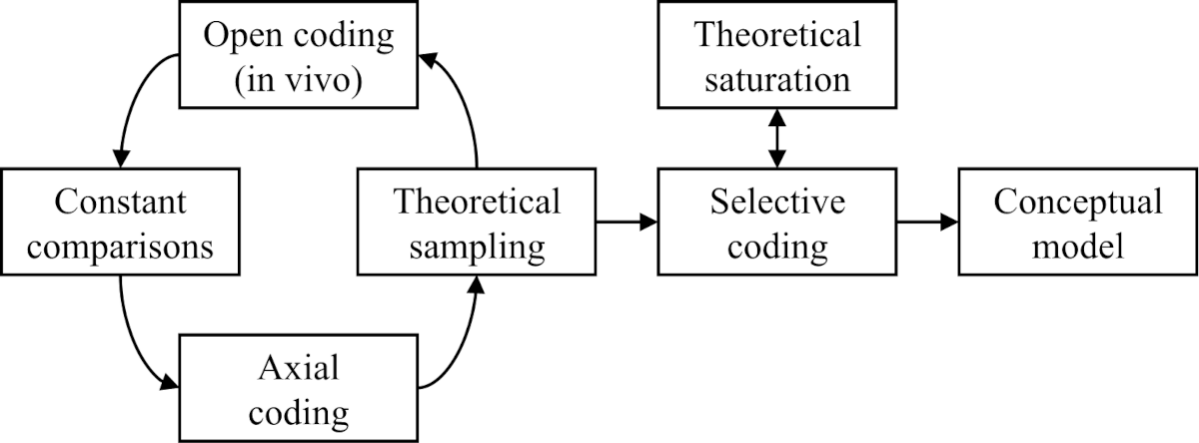
Illustration of the grounded theory process to develop the conceptual model.

For the actual data analysis, the MAXQDA software (version 2020.4.2; VERBI GmbH), a tool for computer-assisted qualitative and mixed methods data analysis [68], was used. The included literature was analyzed line by line to detect statements that refer to digital transformation, health care, and value creation. The first step was open coding, wherein the relevant segments were coded in vivo. An exemplary segment is “[p]atients are facing a multitude of new services and channels to manage their health condition, and new ways of patient interaction might stimulate the occurrence of new business models” [69]. Subsequently, axial coding was used to examine similarities and differences between the generated codes. Similar text segments were aggregated into higher-order categories. During this step, segments such as those aforementioned could be aggregated into several categories, depending on their connection to concepts of relationships or value creation as well as to the identified stakeholder groups. In the case of the exemplary segment, the applied categories include the stakeholder groups of patients and providers as well as categories relating to changes in relationships and patient engagement. In addition, the principles of theoretical sampling were used to enrich the generated data. Theoretical sampling builds on the “all is data” principle in grounded theory, implying that everything the researcher discovers while studying is potentially relevant data [70,71]. It involves inductive reasoning, by generating a general abstraction from particular instances, and determines the selection of concepts and categories that will be further developed over the course of the data analysis [70]. During this step, constant comparisons between the coded segments and the defined higher-order categories resulted in continued revisions of the code structure and its underlying assumptions. After this step, selective coding was used to systematically relate findings to each other and to further refine existing categories into 1 cohesive conceptual model. The text segments were further analyzed quantitatively (ie, frequencies and contingency tables) to assess the relevance of the identified fields. After numerous revisions, a sophisticated level of theoretical saturation was achieved (Figure 2).

**Figure 3:**
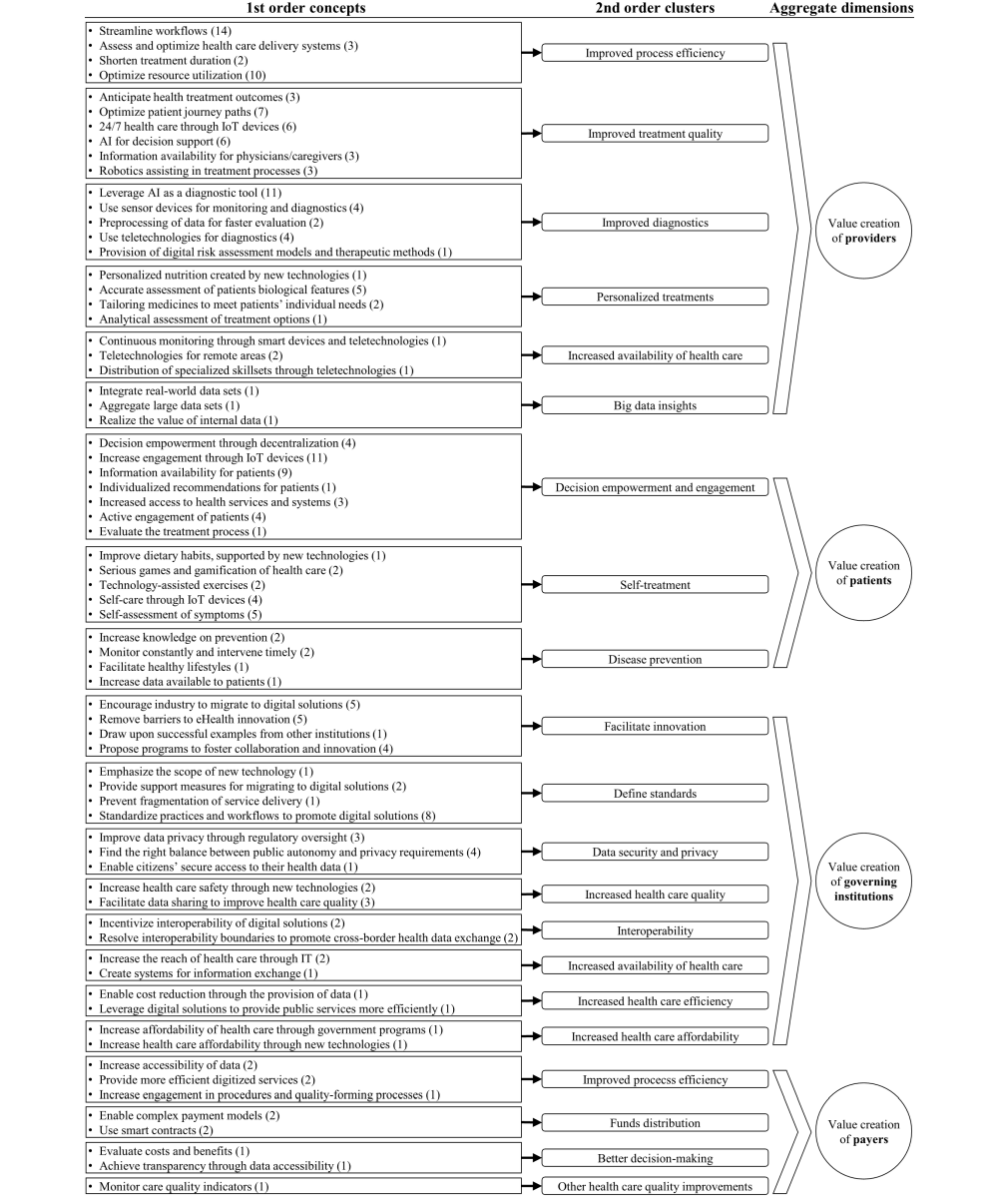
Data structure, with the number of coded segments in parentheses (visualization style adapted from Gioia et al [72]). AI: artificial intelligence; IoT: Internet of Things.

This process resulted in the identification of clusters of value creation for the respective stakeholder groups. Figure 3 provides further insights into the resulting clusters of value creation and the underlying identified concepts in a visualization style adapted from Gioia et al [72].

The same approach was applied to evaluate digital transformation–triggered changes in the relationships among stakeholders. The initial concepts were derived from the identification of text segments involving ≥2 relevant stakeholders. The underlying dynamics describing the changes in the relationships were then aggregated in a similar way as described for the changes in value creation in this section.

## Results

### Overview

The *Results* section is structured as follows. First, the most essential stakeholder groups identified are described. Second, the impact of digital transformation on the value creation of each of the stakeholders is examined. Third, the relationships, shaped by the digital transformation, among the stakeholders are analyzed. A graphical representation of the conceptual model synthesizing the insights is presented in the *Discussion* section.

### Stakeholders in the Health Care Sector

As mentioned in the *Methods* section, the underlying principle of differentiation of the stakeholders was the value they provide for the ecosystem itself or other stakeholders in the system. Consequently, entities representing an organization, such as hospitals, or an individual, such as local physicians, were grouped together. Although they differ by internal processes, they do not differ in terms of their core value proposition for the system in general. Ultimately, 4 stakeholder groups were identified: *governing institutions*, *payers*, *providers*, and *patients*. Table 2 provides an excerpt of the underlying coding structure by presenting the stakeholders and the respective first-level subcategories. The stakeholder groups are defined in the following paragraph.

**Table 2:** Stakeholder groups and first-level subcategories.

Stakeholder group, description or definition, and example				Segment, n
**Governing institutions**				
	**Regulating bodies (both national and international) that define regulatory standards for the health care sector, affecting at least 1 of the other identified stakeholders**			
		Higher order or unspecified	11	
		Government (legislative, executive, and judicial)	5	
**Payers**				
	**Organizations specialized on the reimbursement of providers of medical treatments for the treatment of patients**			
		Insurances	6	
		Higher order or unspecified	6	
		Other payers	3	
**Providers**				
	**Provision of medical treatments to patients by organizations or individual physicians**			
		Medical treatments	16	
		Medical products suppliers	6	
		Higher order or unspecified	5	
		Support functions	3	
		Retail	2	
		Additional services	1	
**Patients**				
	**Receiver of medical treatments**			
		Patients	11	
		Patient advocacy groups	6	
**Other (not considered for research process)^a^**				
	Wider society			13
	Unspecified			2

^a^The 15 segments that could not be matched with the 4 main stakeholders were grouped in the “other” section.

The stakeholder group *governing institutions* considers relevant regulatory bodies (both national and international) that define regulatory standards for the health care sector, affecting at least 1 of the other identified stakeholders. The stakeholder group *payers* refers to organizations specialized in the reimbursement of providers of medical treatments for the treatment of patients. *Providers* focus on the most pivotal type of service, that is, the provision of medical treatments to patients by organizations or individual physicians. Patients are an essential part of the vast majority of stakeholder concepts. For reasons of model complexity and clarity, other groups such as patient advocacy groups were not considered. The stakeholder group *patients* is defined as the receiver of medical treatments.

### Integrative Review

This section presents the results of the integrative review, in particular, on the value creation of stakeholders and the relationships among the stakeholders.

#### Overview of the Results of the Integrative Review

A complete list of all the included literature results is provided in Multimedia Appendix 3 [9,20,21,23,69,73-207], and the design and type of publication are presented in Table 3. The coding process resulted in the coding of 752 text segments in 140 literature results. This extensive collection of data allowed for the identification of initial focal points of the subject area.

**Table 3:** Design and type of the included literature (N=140).

Category			Values, n (%)
**Study design**			
	Qualitative, conceptual research	87 (62.1)	
	Review articles	30 (21.4)	
	Editorials, letters, or viewpoints	16 (11.4)	
	Quantitative research	7 (5)	
**Publication type**			
	Journal article	99 (70.7)	
	Book chapter	21 (15)	
	Conference paper	19 (13.6)	
	Magazine article	1 (0.7)	

#### Value Creation

##### Overview

A co-occurrence analysis shows how often text segments have been assigned certain codes. Looking at both cornerstones of the conceptual model—value creation and relationships—certain trends are observable. The co-occurrence of value creation–related codes with stakeholder codes showed that the value creation of providers (90 segments), followed by patients (50 segments), received the most attention in the literature. Governing institutions (37 segments) and payers (11 segments) were less often addressed in the literature.

##### Value Creation of Providers

The value creation of providers received the most attention in the identified literature. Similar to the effects of digital transformation on process efficiency and quality improvements in manufacturing [208], the coding process showed that the most dominant changes to the value creation of providers in the health care sector are improved process efficiency (29 segments) as well as improved quality of their medical treatments (28 segments). Another relevant aspect is the improvement in diagnostics (22 segments). The ability to provide personalized treatments (9 segments), increased availability of health care (3 segments), and the ability to gain big data insights (3 segments) are relevant aspects as well.

Artificial intelligence (AI) allows for efficiency improvements throughout the treatment process, from admission and diagnostics to treatment and administrative tasks [120,126,166]. More specifically, in combination with other technologies, AI allows for the automated interpretation of injuries and diseases [126,166], accelerates the decision-making process of physicians [82,120], and can automatically modify electronic medical records [166].

Although big data is necessary to enable such a use of AI [126], it also alters the value creation by itself. Big data and information sharing enable the efficient flow of medical information across different providers [150], thus enabling decisions on timely data [133,137], reducing waiting times in subsequent processes [144].

Mobile and wearable devices and apps allow for process efficiency improvements by supporting the workflows of both nursing staff [145] and physicians [144]. In addition, time-consuming activities such as the monitoring of equipment [191] and patients [87,94,179] can be automated.

Improvements in the quality of treatments are mainly attributed to the emergence of AI and big data in medical environments. AI is used along the value chain of medical treatments. It facilitates knowledge management inside the provider organization [114], monitors and evaluates the health status of patients [79,111], proposes recommendations on treatments [128], and supports the decision-making process regarding medical treatments [23,82] and prescriptions [88,133]. This also leads to a reduction in medical errors [116]. The key mechanism is leveraging the knowledge in large data sets for the automated evaluation of current cases. Although AI does not make the final decision, it still greatly supports the physicians in charge.

Without further specifications of the involved technologies, all elements of technology are dynamically connected in a closed loop to continuously improve medical interventions [175], to facilitate new therapeutic procedures [177], and to improve the patient journey [78].

Improvements in diagnostics are primarily related to the advancements and interactions of certain technologies. Technologies such as the Internet of Things, mobile and wearable devices, and telemedicine allow the collection of patient-related data. This can be achieved in an automated fashion via interconnected sensors [122,165,191] or through remote consultations supported by telemedicine technologies [108]. This enables the merging of data sets from different sources [105,122]. Finally, AI applications analyze these data sets to predict and classify health conditions [96,195]. It also enables the large-scale analysis of computed tomography scans and x-rays in an automated fashion [152,185,186].

##### Value Creation of Patients

The digital transformation leads to fundamental changes in the self-conception of patients and how they are engaged in medical decisions and procedures. Thus, patients are increasingly taking an active part in the treatment process. This leverages efficiencies inside the system, which can consequently be characterized as the value creation of patients. In line with changes to the patient-provider relationship, the ongoing empowerment of patients does not necessarily shift value creation away from providers but increases value creation efforts on the patient side. Regarding the coding of segments, 3 distinct clusters could be identified: decision empowerment and engagement (33 segments), self-treatment (14 segments), and disease prevention (6 segments).

Mobile and wearable devices and apps, in addition to the strive of patients to control their health-related tasks [159,192], facilitate the evolution of patients into prosumers, cocreating value with health care providers [157]. Wearable devices allow patients to constantly monitor their health parameters [21,129,177] and communicate relevant insights with their health care providers [150,160]. Integrated decision support systems [186] and personalized recommendations [97] help patients to further evaluate their health status and propose treatment options. The constant availability of monitoring options encourages patients to observe the cause and effect related to their symptoms and treatment procedures [137,147].

Although the literature is often vague about specific technologies involved in the self-treatment process and the process itself [87,100,176], parts of the literature highlight the major role of mobile and wearable devices and apps. These technologies allow patients to self-test for certain conditions [161] and assist in the treatment of conditions. The combination of integrated sensors of these devices allows for an effective monitoring and evaluation, followed by subsequent suggestions for self-treatment activities [23,84,164]. The integration of gamification elements [189] leads to an approach that promotes patient engagement in the process [79].

The interconnectedness of a wide range of digital health technologies [199] encourages patients (or in this case, citizens) to take better care of themselves [190], which further shifts the attention of patients from treatment of diseases to disease prevention [159,192].

##### Value Creation of Governing Institutions

The literature characterizes the value creation of governing institutions as a supporting role for the other stakeholders of the health care sector. The clusters describing the value creation of governing institutions are as follows: facilitate innovation (15 segments), define standards (12 segments), data security and privacy (8 segments), increased health care quality (5 segments), interoperability (4 segments), increased availability of health care (3 segments), increased health care efficiency (2 segments), and increased health care affordability (2 segments).

The changing value creation of governing institutions is closely linked to network-like collaboration systems in the provider–governing institutions relationship (refer to the *Provider–Governing Institutions Relationship* subsection). In general, governing institutions are expected to play a more active role in the further optimization efforts of the health care sector [130]. As new business models emerge that challenge existing regulatory frameworks [135], participants in the health care sector are limited in their innovation aspirations by strict regulations [207]. One of the reasons for this is the lack of capabilities and capacities of the respective institutions to integrate innovations fast enough into regulations [20,183]. Consequently, the role of governing institutions in modern health systems exceeds that of evaluating specific technological solutions. Instead, the role shifts toward creating incentives that foster collaboration [140] and steer the development and adoption of technological solutions that contribute to effective health systems [158,196].

To enable the creation of effective health systems, interoperability has to be ensured, which requires the definition of certain standards and data security and privacy regulations. Consistent standards help to guarantee functioning solutions [92,193] and drive all actors toward the holistic vision of the health care system [168]. It also reduces the risk of investments in digital solutions of other participants in the health care sector [202]. Regarding patients, a lack of standards may lead to decreased trust in the system [188]; thus, privacy concerns may delay the adoption of digital solutions [96,136,139]. Consistent standards also enable the ability to address the global health goals [206] by supporting real-time health data assessment across national borders [204]. In general, governing institutions must achieve the right balance between public autonomy and privacy requirements [115] to support value creation within the health care sector.

##### Value Creation of Payers

The increasingly connected relationship between payers and providers, for example, enabled by digital platforms, allows payers to become involved in the processes of providers [197]. Digitalized processes and data allow for efficiency increases [149], such as accelerating the processing of insurance claims [86,151]. Increased connectivity also facilitates the adoption of flexible payment systems, designed to accommodate for specified outcomes, across multiple health care settings [118]. Technologies such as blockchain are even capable of automating the distribution of funds via smart contracts while ensuring immutable data trails that lead to better fraud detection and overall better decision-making [174,187]. The identified clusters of value creation are as follows: increased process efficiency (5 segments), fund distribution (4 segments), better decision-making (2 segments), and overall health care quality improvements (1 segments).

#### Relationships

##### Overview

The analysis of relationship-coded segments shows how often text segments are assigned a relationship-related code and at least 2 stakeholder codes (Table 4). The patient-provider relationship received the most attention in the literature, accounting for almost one-third (39/120, 32.5%) of all assigned relationship codes. On an individual stakeholder level, the relationships of providers with others receive the most attention (54/120, 45% segments) and those of governing institutions receive the least attention (9/120, 7.5% segments).

**Table 4:** Co-occurrence of stakeholders in relationship-coded text segments (n=120).

	Patients (number of co-occurrences), n	Providers (number of co-occurrences), n	Payers (number of co-occurrences), n	Governing institutions (number of co-occurrences), n	Segment, n (%)
Patients	N/A^a^	39	5	0	44 (36.7)
Providers	39	N/A	7	8	54 (45)
Payers	5	7	N/A	1	13 (10.8)
Governing institutions	0	8	1	N/A	9 (7.5)

^a^N/A: not applicable.

##### Patient-Provider Relationship

The patient-provider relationship under digital transformation received the most attention in the literature. It is shaped by 3 key findings: initiating contact, equal partnerships, and weakening of direct links.

Technological advancements facilitate the initiation of the patient-provider contact. In particular, telemedicine removes physical and mental barriers and allows patients to make better choices regarding preferred providers [84,141,197]. Advancements in information and communications technology have simplified the process of booking an appointment [95,99,209].

The most common change is related to the strengthening of the patient-provider relationship. Increased access to one’s own medical information allows patients to communicate in an effective [101,113,115,162,175] and safe [156] way with providers. This also enables patients to be more engaged in the treatment process [110,129,160]. In addition, optimizations in providers’ workflows allow them to spend more time with patients [201]. Ultimately, patients and providers will become equal partners in the treatment process to make medical decisions [123,182].

Advancements in platform developments have reached the medical field. This redefines the link between patients and providers. Platforms play an active role through the allocation of data from various sources and subsequent processing. Owing to the large number of potential stakeholders, platforms distribute the data according to patients’ preferences [124]. As a consequence, large parts of the patient-provider interactions are facilitated and managed by intermediaries such as platforms, weakening the direct link between patients and providers [23,69,86,95].

##### Patient-Payer Relationship

Only a fraction of the literature discusses the patient-payer relationship. However, digital technologies enable an easier exchange of information between patients and payers [111]. The interactions between these 2 stakeholder groups are predominantly simplified [194] through the help of intermediaries such as digital platforms [86]. This further facilitates the desire of patients to be involved in the decision-making processes of payers regarding adequate interventions and diagnosis [197].

##### Patient–Governing Institutions Relationship

Although the relationships between patients and governing institutions in our society are apparent, no text segments indicating substantial digital transformation–related changes to the patient–governing institutions relationship could be identified.

##### Provider-Payer Relationship

The provider-payer relationship is mainly characterized by an increasing connectivity, leading to closer collaborations. This connectivity does not necessarily have to be a direct link but can be achieved through an intermediary, orchestrating the distribution of data [86], and is motivated by the desire to reduce barriers and costs [73,111,175]. However the relationships are managed, novel technologies such as blockchain facilitate the strengthening of relationships by allowing for higher trust in the transparency and security of the system [194], as well as efficiency improvements [187], needed for the provision of innovative care models [202].

##### Provider–Governing Institutions Relationship

The provider–governing institutions relationship is characterized by the exploitation of network capabilities. The ongoing interconnectedness of these 2 stakeholders bolsters collaboration and causes more linkages that require closer relationships. This is shown by the construction of political and legislative guidelines, where both parties rest together to determine the terms [135]. This leads to the appropriate design of network-like collaboration systems [73], where governing institutions usually take the role of the resource allocator [118,119,171]. Other types of collaborations are characterized by structured transformation programs that require a close relationship between these 2 stakeholders [140,169].

##### Payer–Governing Institutions Relationship

There are no indications in the identified literature that the payer–governing institutions relationship is influenced substantially by digital transformation. Changes to this relationship are mainly characterized by the participation of these 2 groups in health care ecosystems, where information is exchanged [73].

## Discussion

### Conceptual Model of the Health Care Sector Under Digital Transformation

#### Unifying Perspective on Health Care

**Figure 4:**
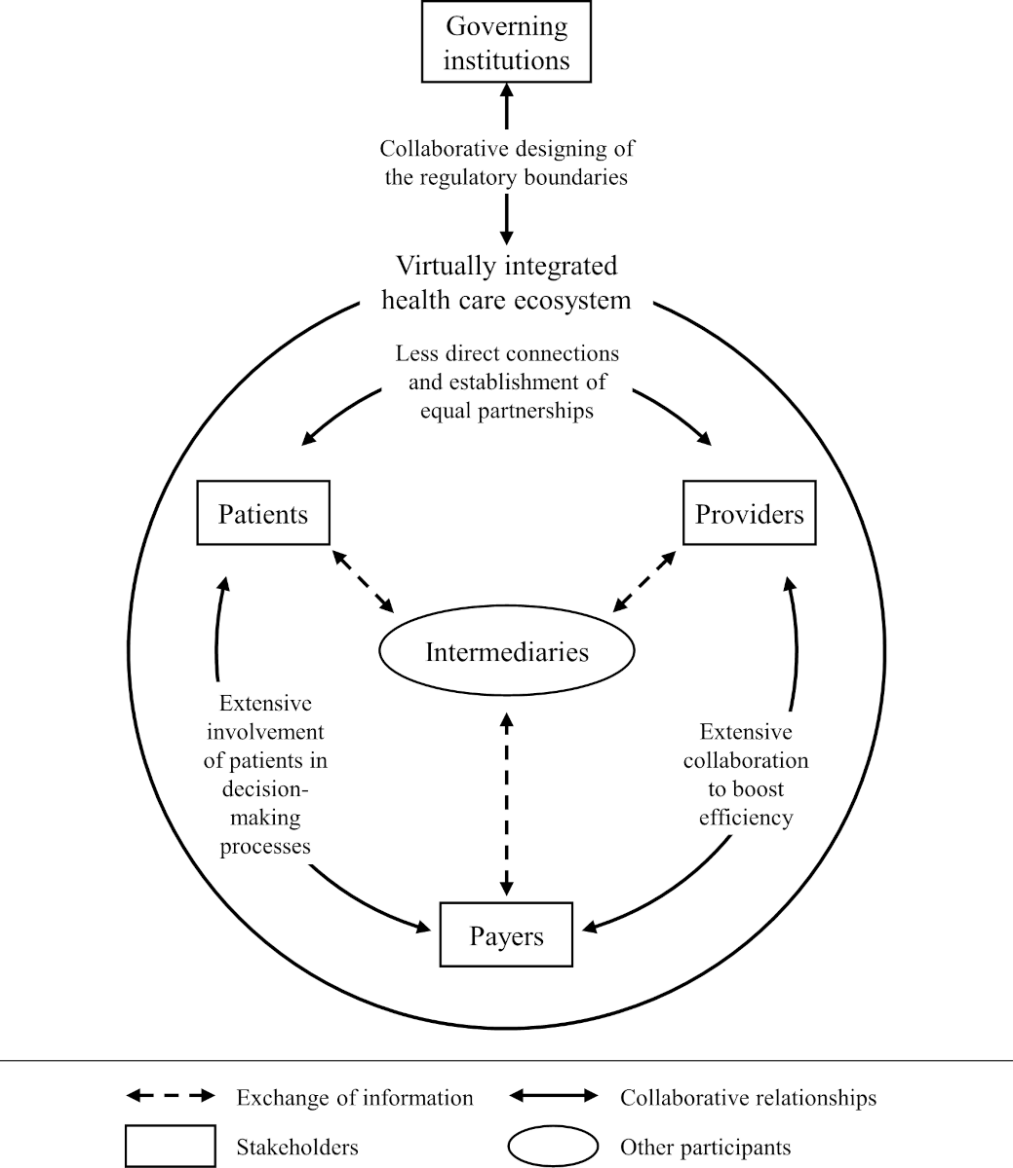
Conceptual model of the health care sector under digital transformation.

The research process allowed for the constant integration of new findings and was neither limited to the elaborations in the *Introduction* section nor to the findings in the *Results* section. The following sections synthesize the in-depth view on the impact of digital transformation on the health care sector and derive a visual representation of a conceptual model (Figure 4). The model exemplifies the need to consider other participating entities and the need for a holistic perspective on the collective actions of participants in the health care sector. The following sections elaborate the characterizations of the new elements introduced in the model.

#### Intermediaries in the Health Care Sector

During the research process, the need to consider participants other than the 4 stakeholders, identified from the existing stakeholder models, emerged. As described in the *Relationships* subsection in the *Results* section, intermediaries play an important role in managing and distributing relevant data toward different entities [128,197].

However, the literature does not yet provide a clear conceptualization of these intermediaries. The necessity of these intermediaries predominantly stems from the financial and technological limitations of existing players in the health care sector to develop technology-based health solutions [69,103]. Specifically, other stakeholders are limited in their capabilities to process large amounts of data [103] or even gather data from multiple sources in the health care sector. With advanced data collection, processing, and preparation capabilities [21,164], intermediaries are able to cross-link information to derive new insights for various actors in the sector [198]. As a result, value creation becomes more modular and additive, allowing for solutions tailored to specific use cases [69,157].

The literature often referred to platforms [69,86,95] or ecosystems [73,161] as necessary entities to orchestrate this exchange of data. However, the conflicting interests of the societal perspective of research for the common good based on health data and the individuals’ perspective of privacy concerns and fair compensation for the donation of data are more relevant than ever [155,210]. The health care industry is already moving toward complex interacting multisided markets [157] to substitute the potentials of a much-needed connected digital infrastructure across borders [73].

Discussions on the appropriate conceptualizations of these types of intermediaries are therefore intensifying. To avoid the well-known monopolistic tendencies in such constructs [69], various initiatives are fostering discussions on other conceptualizations of intermediaries. Data spaces and federated data ecosystems in health care [211,212] are the driving force behind discussions regarding the consideration of health care data as common good or public asset [213] and data privacy, sovereignty, and trust [118,214]. The current dynamics in this field do not permit a precise specification of these intermediaries, as new research streams are adding even more ambiguity. Consequently, this model can serve as a starting point for future specifications of these intermediary entities.

#### The Virtually Integrated Health Care Ecosystem

The shift from linear value chains to multisided markets requires a different perspective on the health care sector. The creation of value for different stakeholders increasingly occurs through complex collaborations among stakeholders, sometimes provisioning services that could not be offered otherwise [119]. The cocreation of value is enabled by interconnected systems [161] and the continuous exchange of information within the network [155,172]. This exemplifies the need for a holistic perspective on health care ecosystems to ensure the necessary coordination of various actors according to common goals [73,144].

The term “virtually integrated health care ecosystem” illustrates the dualism emerging in the health care sector. Certainly, tighter relationships and coordinated efforts typically increase efficiency by driving specialization in the value network. However, extensive sharing of information allows for the collective creation of personalized modular values for actors inside the system. Virtual integration differs from other arrangements with partners by treating them as if they were inside the organization [215,216]. The term “virtual integration” was originally coined by Michael Dell, describing similar benefits from a business model perspective [215]. However, the health care sector with its low digital transformation maturity level and the high level of dynamism of digital transformation could cause distortions that challenge the viability of the concept of a virtually integrated health care ecosystem.

### Principal Findings

This study systematizes the relevant academic literature at the intersection of digital transformation and health care. The aim was to provide a conceptual model, giving a holistic view on the sector that goes beyond the technological aspects. The results were obtained from a systematic literature search. The aggregation of qualitative data was conducted using a grounded theory approach, which is an inductive and systematic data analysis methodology that yields strong descriptive and explanatory power at the intersection of theory and data [67]. We provide an in-depth overview of the relevant literature through the lenses of value creation of, and relationships among, stakeholders. The conceptual model highlights the interdependencies of various stakeholders in the health care sector. In addition, digital transformation forces stakeholders to adapt an ecosystem way of operating, accepting the principles of value cocreation and the central role of intermediaries.

### Implications

From a theoretical point of view, the results are intended to guide further discussions on digital transformation in health care. To the best of our knowledge, no other publications conceptualizing a holistic view on the health care sector under digital transformation exist.

However, other scholars have also identified the crucial role of digital technologies or digital capabilities in supporting value creation innovation in health care. In intraorganizational settings, Gopal et al [20] described the interplay among intraorganizational data providers, the generation of insights through advanced technologies, and the exploitation of these insights in business processes. Ghosh et al [217] highlighted the role of information technology capabilities in creating new value propositions inside organizations. An imperative element is the ability to integrate new data into the organization. In a home-based recovery setting, Dimitrov [128] highlighted the need for an entity to distribute information across entities inside the health care systems. From a microlevel perspective, these conceptualizations implicitly highlight the need for interorganizational data exchange and the lack of respective capabilities of the current health care stakeholders to orchestrate such an exchange.

Other streams of research in information systems literature consider the workforce transformation [218] or the transformation process of organizational change [219], both driven by technological change. Although the microlevel perspective is common for transformation process conceptualizations, it is worth considering that key elements such as the impact of technologies on the external and internal dimensions of organizations are key elements of these models as well.

Therefore, the proposed conceptual model of this study follows the commonly agreed upon key drivers and dimensions of digital transformation. However, it provides a novel perspective on the interrelations among the actors in the health care sector. Therefore, it can be the foundation of further evaluations of the strategic actions of actors and their effects on other actors or the health care ecosystem itself. Specifically, in the context of ecosystem-enabled value creation activities, the model can guide paths to answer questions of interest at the scientific level.

The conceptual model introduced intermediaries as participants in the health care ecosystem. In this model, these intermediaries are mainly related to the exchange of information within the system. The literature, however, views these intermediaries as a heterogeneous group, for example, consisting of so-called big tech companies [164], start-ups [21,198], or unspecified actors [118]. The reduction of this heterogeneous group to the core value it introduces into the sector strengthens the generalizing perspective of this study. Considering that this study aims to lay the groundwork for a unified perspective on the digital transformation in the health care sector, this allows for further examinations of specific parts of the model in the future.

From a practical point of view, providers are becoming increasingly dependent on intermediaries as essential parts of the value creation and patient interaction are enabled by outside actors. Providers might face a lock-in effect when intermediaries manage to establish de facto standards that are outside of the providers’ control. To avoid future negative strategic positions, providers are encouraged to establish their own intermediary entities to maintain control over the essential parts of the patient journey.

Patients are experiencing a growing influence in the health care sector. With technology allowing a plethora of ways to provide and evaluate value, patients are becoming more selective in their decisions. Skepticism regarding data privacy and protection forces other actors to pay special attention to the interests of patients. Technology-enabled empowerment of patients not only levels the playing field between individual patients and providers but also increases the influence of patient advocacy groups [220,221]. These groups might strive to advance into an intermediary position to take control of the information distribution process, in the best of the patients’ interests.

Payers, being part of the data-driven insurance industry, also have a strong interest to grow their influence on intermediaries to exploit the enormous amounts of data for risk and benefit evaluations. Simultaneously, payers might encounter opportunities and threats from emerging technologies such as blockchain. Although blockchain provides exclusive opportunities for fraud detection, smart contracts are able to challenge payers’ business models. In particular, there is an increasing connection among the actors. Although extensive regulations in the health care sector might impede fully automated processes based on smart contracts, payers are dared to ignore this technological trend.

Accordingly, elaborations should not be limited to single actors but should view the health care sector as a system of value cocreation. Policy makers are encouraged to pay special attention to possible interdependencies in the complex system of stakeholders when proposing policy decisions. For these decisions, they are going to see themselves negotiating with changing compositions of interest groups from the virtually integrated health care ecosystem. This increases the difficulty of introducing effective governance mechanisms and incentive structures to steer stakeholders’ initiatives. Policy makers should also pay special attention to intermediaries in the health care sector and avoid leaving these developments exposed to unrestricted market forces. As Bates et al [222] describe it, public policy is simultaneously the key enabler and the key barrier to unleashing the potential of health care data [222]. Regulators must determine the scope of information sharing and linking, considering the interest of various groups in the health care sector.

Policy makers are also advised to closely monitor the sector to detect de facto monopolies in certain key areas as consequences of critical mass effects [223]. Mandatory open standards and interfaces are able to limit the influence of single entities and allow for the emergence of new entrants. The digital transformation–induced disruptions require metrics that enable policy makers to assess the overall effectiveness of the health care sector. In this regard, future research should develop adequate key performance indicators. These key performance indicators must be adequately designed to detect possible inefficiencies and monopolistic activities in key areas, even in small segments of the sector. Special attention should be paid to the multidimensional nature of digital platforms, which poses new challenges with regard to the assessment of their impact on the market. In particular, policy makers in the European market are advised to monitor this segment, as dominating digital platforms are currently based in either the United States or China [224].

### Limitations

The very nature of the integrative literature approach leads to possible limitations for this study. First, the search strategy has an impact on the results. In this study, the search results were classified as digital transformation related or Healthcare 4.0 related by the respective authors. It is possible that more abstract search terms (eg, referring to specific technologies, stakeholders, or actions of stakeholders) could yield different results. It is also possible that the term “digital health” would have yielded additional relevant results. However, owing to the broad scope of this term and the lack of association with the fundamentals of digital transformation in academic discussions, we decided to not consider this term in our search. As our approach yielded more relevant records than other works, we do not regard this search strategy as too narrow [157,225].

Given the novelty of this study, the sufficiency of the data involved in the research process was judged by 2 measures, the data basis of other studies and “theoretical saturation” [45]. This study included 140 literature results in the data evaluation, which is well above the number of publications included in other studies. For example, Kraus et al [31] evaluated the current state of research of digital transformation in health care and included 27 articles; the work of Kutnjak et al [225], analyzing the effect of digital transformation in health care in practice, included 7 case studies; Hermes et al [157] investigated the digital transformation of the health care industry based on 110 literature results; Marques and Ferreira [180] used 53 articles to analyze the emerging trend of digital medicine; and da Silveira et al [122] included 10 research articles to systematize and qualitatively describe the contributions of Industry 4.0 in the context of the health sector.

Looking at the theoretical saturation, the number of codes applied to text segments was >700. During the codification process, the most relevant code clusters emerged after approximately one-third of the literature was coded. The following research process further strengthened the results, rather than challenging it. This effect was also facilitated by the aim of this study to propose a conceptualization of the health care sector, which puts more emphasis on fundamental concepts rather than individual findings. On the basis of these 2 measures, we are confident that this study provides a sufficient coverage of the relevant literature.

Second, it is possible that the full ramifications of digital transformation can only be recognized retrospectively in the future. Future research might challenge the view of recent literature that concepts such as changing value creation and relationships are essential for digital transformation and propose different determining factors of the transformation we are currently experiencing.

Third, the grounded theory–based coding methodology itself, although followed rigorously, might rely on subjective perceptions of the researchers, thereby affecting the results.

The decisions made during the stakeholder identification also have considerable implications on the methodology and results of this study. Scholars providing in-depth analyses of the health care sector limit their perspective to comparable numbers of stakeholders [31,57,58]. Others, listing numerous stakeholders, do not provide such in-depth analyses [48-55]. In line with the aim of this study, we limited our perspective to the most essential stakeholders to be able to provide in-depth analyses of the complex interrelations occurring in the health care sector. However, an in-depth examination of the heterogeneous group intermediaries could lead to the identification of various other important stakeholders and further differentiate various means of value creation. In addition, future research should consider the integration of possible contributions of other stakeholder groups disregarded for the purposes of this study. Stakeholders in academia and research could advance the conceptual model and lead to an even more differentiated view on the health care sector under digital transformation. Furthermore, the lack of differentiation of the level of analysis within the individual stakeholder groups may limit the findings. For example, the *providers* group includes both individuals (eg, physicians) and organizations (eg, hospitals). The different characteristics of these entities may have implications for value creation or relationships that were not further differentiated in this study to address the need for ecosystem-level abstraction.

### Conclusions

The proposed conceptual model highlights the interconnectedness of stakeholders’ digital transformation facilitates in the health care sector. We introduced the concept of the virtually integrated health care ecosystem and elaborated on the central role of intermediaries inside the system. Stakeholders are encouraged to recognize the common goal of actors in the sector and to embrace novel forms of cooperation. The proposed model can therefore serve practitioners as a useful tool to evaluate strategic responses. In addition, policy makers are advised to recognize the emerging reality inside the health care sector and let it reflect on their policies. Finally, it provides researchers with a basis to guide further scientific discussions on the transformation of the health care sector.

## Appendix

Multimedia Appendix 1 PRISMA-ScR (Preferred Reporting Items for Systematic Reviews and Meta-Analyses extension for Scoping Reviews) checklist.

Multimedia Appendix 2 Database search terms.

Multimedia Appendix 3 Literature included in the integrative review.

## Abbreviations

AI: artificial intelligence
MECE: Mutually Exclusive and Collectively Exhaustive
PRISMA: Preferred Reporting Items for Systematic Reviews and Meta-Analyses

## Data Availability

The data sets generated or analyzed during this study are available from the corresponding author upon reasonable request.
