# Supplementary material for: Development of an Evidence-Based Conceptual Model of the Health Care Sector Under Digital Transformation: Integrative Review
Source: J Med Internet Res. 2023 Jun 8;25:e41512. doi: 10.2196/41512 (PMC10288351; doi:10.2196/41512)
Supplement: Multimedia Appendix 2 [file jmir_v25i1e41512_app2.pdf]

Multimedia Appendix 2

This is a Multimedia Appendix to a full manuscript published in the J Med Internet Res.
For citation information see http://doi.org/10.2196/41512

Table 1: Database search terms

**database**	**search term**
Web of Science	AB=("Health 4.0" OR "Healthcare 4.0" OR "Health Care 4.0" OR ("Digital Transformation" AND (Health*))) OR TI=("Health 4.0" OR "Healthcare 4.0" OR "Health Care 4.0" OR ("Digital Transformation" AND (Health*)))
PubMed	("Health Care 4.0"[Title/Abstract]) OR ("Healthcare 4.0" [Title/Abstract]) OR ("Health 4.0"[Title/Abstract]) OR (("Digital Transformation"[Title/Abstract]) AND ((Health*[Title/Abstract])))
dimensions.ai (search in: "Title and Abstract" selected)	("Health 4.0" OR "Healthcare 4.0" OR "Health Care 4.0" OR ("Digital Transformation" AND (Health)))
