# Supplementary material for: Development of an Evidence-Based Conceptual Model of the Health Care Sector Under Digital Transformation: Integrative Review
Source: J Med Internet Res. 2023 Jun 8;25:e41512. doi: 10.2196/41512 (PMC10288351; doi:10.2196/41512)
Supplement: Multimedia Appendix 3 [file jmir_v25i1e41512_app3.pdf]

Multimedia Appendix 3

This is a Multimedia Appendix to a full manuscript published in the J Med Internet Res.
For citation information see http://doi.org/10.2196/41512

Table 1: Literature included in the integrative review

Author (year)	Title
Aarestrup et al. (2020)	Towards a European health research and innovation cloud (HRIC)
Abrahams and Matusheski (2020)	Personalised nutrition technologies: a new paradigm for dietetic practice and training in a digital transformation era
Abugabah et al. (2020)	Decentralized Telemedicine Framework for a Smart Healthcare Ecosystem
Aceto et al. (2020)	Industry 4.0 and Health: Internet of Things, Big Data, and Cloud Computing for Healthcare 4.0
Agarwal et al. (2010)	Research Commentary —The Digital Transformation of Healthcare: Current Status and the Road Ahead
Agrawal (2020)	Bridging digital health divides
Ahmad et al. (2022)	Architecting Intelligent Smart Serious Games for Healthcare Applications: A Technical Perspective
Ahmadi-Assalemi et al. (2020)	Digital Twins for Precision Healthcare
Akatkin et al. (2017)	Digital economy: Conceptual architecture of a digital economic sector ecosystem
Al-Aswad et al. (2021)	BZKP: Blockchain-based zero-knowledge proof model for enhancing healthcare security in Bahrain IoT smart cities and COVID-19 risk mitigation
Alauddin et al. (2021)	The Modern and Digital Transformation of Oral Health Care: A Mini Review
Alcan (2020)	Internet of Things (IoT) in Healthcare Systems
Alghamdi (2019)	Monitoring Mental Health Using Smart Devices with Text Analytical Tool
Al-Jaroodi et al. (2020)	Health 4.0: On the Way to Realizing the Healthcare of the Future
Amruthamma and Ashwatha Narayana (2017)	Indian Health Care and Insurance Industry on the Eco-System of a Digital Transformation

-continued-

**Author (year)**	**Title**
Anwar et al. (2015)	Anytime, anywhere access to secure, privacy-aware healthcare services: Issues, approaches and challenges
Arabi et al. (2021)	How the COVID-19 pandemic will change the future of critical care
Arpaia et al. (2021)	Design, implementation, and metrological characterization of a wearable, integrated AR-BCI hands-free system for health 4.0 monitoring
Asthana et al. (2019)	Why does the NHS struggle to adopt eHealth innovations? A review of macro, meso and micro factors
Atreja et al. (2019)	Digital Medicine and Evolution of Remote Patient Monitoring in Cardiac Electrophysiology: A State-of-the-Art Perspective
Auerbach et al. (2020)	Letting a Good Crisis Go to Waste
Aujla et al. (2019)	DLRS: Deep Learning-based Recommender System for Smart Healthcare Ecosystem
Awad et al. (2021)	Connected healthcare: Improving patient care using digital health technologies
Baby and Chandran (2022)	Digital Health Infrastructure
Backholer et al. (2021)	Chapter 6 Digital determinants of health: the digital transformation
Bagaria et al. (2020)	Health 4.0: Digital Twins for Health and Well-Being
Barnett et al. (2019)	Digital transformation of hospital quality and safety: real-time data for real-time action
Basatneh et al. (2018)	Health Sensors, Smart Home Devices, and the Internet of Medical Things: An Opportunity for Dramatic Improvement in Care for the Lower Extremity Complications of Diabetes
Baudin et al. (2020)	Views of Swedish Elder Care Personnel on Ongoing Digital Transformation: Cross-Sectional Study
Bause et al. (2019)	Design for Health 4.0: Exploration of a New Area
Belliger and Krieger (2018)	The Digital Transformation of Healthcare

-continued-

**Author (year)**	**Title**
Benis et al. (2021)	One Digital Health: A Unified Framework for Future Health Ecosystems
Benjamin and Potts (2018)	Digital transformation in government: Lessons for digital health?
Bhardwaj et al. (2020)	Mobile FOG Architecture Assisted Continuous Acquisition of Fetal ECG Data for Efficient Prediction
Bhattacharya et al. (2021)	BinDaaS: Blockchain-Based Deep-Learning as-a-Service in Healthcare 4.0 Applications
Bhatti et al. (2019)	Recommendation system using feature extraction and pattern recognition in clinical care systems
Biancone et al. (2021)	E-health for the future. Managerial perspectives using a multiple case study approach
Bin Ahmad et al. (2022)	Emerging trends and evolutions for smart city healthcare systems
Bonacina et al. (2021)	Can the European EHR Exchange Format Support Shared Decision Making and Citizen-Driven Health Science?
Bongaerts et al. (2017)	The Pac-Man Principle in the Healthcare Market
Busse et al. (2021)	Views on Using Social Robots in Professional Caregiving: Content Analysis of a Scenario Method Workshop
Cáceres et al. (2019)	Towards Health 4.0: e-Hospital Proposal Based Industry 4.0 and Artificial Intelligence Concepts
Caumanns (2019)	For discussion: The state of digitization of the German healthcare system
Cavallone and Palumbo (2020)	Debunking the myth of industry 4.0 in health care: insights from a systematic literature review
Cerchione et al. (2022)	Blockchain’s coming to hospital to digitalize healthcare services: Designing a distributed electronic health record ecosystem
Choi et al. (2018)	Subscribing to Your Patients — Reimagining the Future of Electronic Health Records
Chute and French (2019)	Introducing Care 4.0: An Integrated Care Paradigm Built on Industry 4.0 Capabilities
Colombo et al. (2021)	Health Information Systems, Electronic Medical Records, and Big Data in Global Healthcare

-continued-

Author (year)	Title
Corneliussen (2020)	Developing a regional solution to digital transformation of health care services: Collaboration, leadership, and technology expertise
Cresswell et al. (2021)	Interorganizational Knowledge Sharing to Establish Digital Health Learning Ecosystems: Qualitative Evaluation of a National Digital Health Transformation Program in England
Crisan and Mihaila (2021)	Health-care information systems adoption – a review of management practices
Da Silveira et al. (2019)	Analysis of Industry 4.0 Technologies Applied to the Health Sector: Systematic Literature Review
Darmawan and Laksono (2021)	The New Leadership Paradigm in Digital Health and Its Relations to Hospital Services
Dassel and Klein (2020)	(My) data for (my) health – privacy calculi of terminally-ill patients with rare diseases
Datta (2016)	Retail Medicine in an Era of IoT and Medical Errors in the Age of Ubiquitous Connectivity
Delgado (2021)	Without IPv6, there is no digital transformation for healthcare
Diamantopoulos et al. (2019)	Secure Cross-Border Exchange of Health Related Data: The KONFIDO Approach
Dimitrov (2016)	Medical Internet of Things and Big Data in Healthcare
Ding et al. (2019)	Digital health for COPD care: the current state of play
do Nascimento et al. (2020)	Covid-19: A Digital Transformation Approach to a Public Primary Healthcare Environment
Dossou et al. (2021)	Intelligent Support System for Healthcare Logistics 4.0 Optimization in the Covid Pandemic Context
Edberg and Wendel (2018)	Healthcare Transformation: The Electronic Health Record
Eden et al. (2020)	Digitising an Australian university hospital: qualitative analysis of staff-reported impacts
Efendy et al. (2022)	National Health Insurance (JKN) Mobile Application Use Towards Satisfaction of Participants of the Health Social Security Implementing Agency (BPJS) in Madani Hospital in Medan City

-continued-

Author (year)	Title
Ekeland and Linstad (2020)	Elaborating Models of eHealth Governance: Qualitative Systematic Review
El Majdoubi et al. (2021)	SmartMedChain: A Blockchain-Based Privacy-Preserving Smart Healthcare Framework
Eriksen et al. (2020)	The Digital Transformation of Patient-Reported Outcomes' (PROs) Functionality Within Healthcare
Estrela et al. (2019)	Health 4.0: Applications, Management, Technologies and Review
Evangelatos et al. (2020)	Digital Transformation and Governance Innovation for Public Biobanks and Free/Libre Open Source Software Using a Blockchain Technology
Faddis (2018)	The Digital Transformation of Healthcare Technology Management
Fischer (2020)	Service Innovation in Health Care: The Role of Health Platforms as Innovators
Flórez et al. (2020)	Application of Automation and Manufacture techniques oriented to a service-based business using the Internet of Things (IoT) and Industry 4.0 concepts. Case study: Smart Hospital
Frączkiewicz-Wronka (2021)	Digitalization of healthcare sector as a tool for implementation of competition policy
Francis Gomes et al. (2017)	Towards business ecosystems for connected health
Frennert (2021)	Gender blindness: On health and welfare technology, AI and gender equality in community care
Gadupudi et al. (2017)	Teledentistry: A futuristic realm of dental care
Galetsi et al. (2022)	Assessing Technology Innovation of Mobile Health Apps for Medical Care Providers
Gasova and Stofkova (2017)	E-Government as a Quality Improvement Tool for Citizens' Services
Gavrilov et al. (2020)	Analysis of Digitalization in Healthcare: Case Study
Gibson (2016)	The eRedbook: Enabling digital transformation in health care

-continued-

Author (year)	Title
Gleiss et al. (2021)	An apple a day – how the platform economy impacts value creation in the healthcare market
Gochhait et al. (2020)	Implementation of EHR using Digital Transformation: A study on Telemedicine
Gökalp et al. (2021)	Leveraging Digital Transformation Technologies to Tackle COVID-19: Proposing a Privacy-First Holistic Framework
Gopal et al. (2019)	Digital transformation in healthcare - architectures of present and future information technologies
Grigoriadis et al. (2016)	Health 4.0: The Case of Multiple Sclerosis
Gupta et al. (2020)	VAHAK: A Blockchain-based Outdoor Delivery Scheme using UAV for Healthcare 4.0 Services
Hahn and Schreiber (2019)	E-Health
Hasselgren et al. (2021)	Blockchain for Increased Trust in Virtual Health Care: Proof-of-Concept Study
Hermes et al. (2020)	The digital transformation of the healthcare industry: exploring the rise of emerging platform ecosystems and their influence on the role of patients
Herrero-Martinez et al. (2022)	Dynamic Regulatory Assessment: evolving the European Regulatory Framework for the Benefit of Patients and Public Health-an EFPIA View
Ianculescu and Alexandru (2020)	Microservices - A Catalyzer for Better Managing Healthcare Data Empowerment
Ilin et al. (2022)	Critical Factors and Challenges of Healthcare Digital Transformation
Iyamu et al. (2021)	Defining Digital Public Health and the Role of Digitization, Digitalization, and Digital Transformation: Scoping Review
Jahankhani and Kendzierskyj (2019)	Digital Transformation of Healthcare
S.-J. Kim et al. (2020)	Current State and Strategy for Establishing a Digitally Innovative Hospital: Memorial Review Article for Opening of Yongin Severance Hospital
S. E. Kim et al. (2021)	Digital Transformation in Ophthalmic Clinical Care During the COVID-19 Pandemic

-continued-

**Author (year)**	**Title**
Kishor and Chakraborty (2021)	Artificial Intelligence and Internet of Things Based Healthcare 4.0 Monitoring System
Klinker et al. (2020)	Digital Transformation in Health Care: Augmented Reality for Hands-Free Service Innovation
Kotz et al. (2016)	Privacy and Security in Mobile Health: A Research Agenda
Kouroubali and Katehakis (2019)	The new European interoperability framework as a facilitator of digital transformation for citizen empowerment
Krasuska et al. (2021)	Driving digital health transformation in hospitals: a formative qualitative evaluation of the English Global Digital Exemplar programme
Kumar et al. (2020)	A Novel Smart Healthcare Design, Simulation, and Implementation Using Healthcare 4.0 Processes
Lehne et al. (2019)	Why digital medicine depends on interoperability
Leone et al. (2021)	How does artificial intelligence enable and enhance value co-creation in industrial markets? An exploratory case study in the healthcare ecosystem
Lerzynski (2021)	Opening the Door for Digital Transformation in Hospitals: Management’s Point of View
H. Li et al. (2018)	Blockchain-Based Data Preservation System for Medical Data
J. Li and Carayon (2021)	Health Care 4.0: A Vision for Smart and Connected Health Care
J.-P. O. Li et al. (2021)	Digital technology, tele-medicine and artificial intelligence in ophthalmology: A global perspective
Loch et al. (2021)	Digital transformation in urology-opportunity, risk or necessity?
Lux et al. (2017)	Digitalisierung im Gesundheitswesen — zwischen Datenschutz und moderner Medizinversorgung
Manogaran et al. (2017)	Big Data Security Intelligence for Healthcare Industry 4.0
Marques and Ferreira (2020)	Digital transformation in the area of health: systematic review of 45 years of evolution

-continued-

**Author (year)**	**Title**
McEachern and Cholewa (2017)	Digital Health Services and Digital Identity in Alberta
Meskó (2014)	Rx Disruption: Technology Trends in Medicine and Health Care
Meskó et al. (2017)	Digital health is a cultural transformation of traditional healthcare
Mukherjee and Singh (2020)	The Opportunities of Blockchain in Health 4.0
Mun et al. (2020)	Artificial Intelligence for the Future Radiology Diagnostic Service
Müschenich and Wamprecht (2018)	Health 4.0 - how are we doing tomorrow?
Narikimilli et al. (2020)	Blockchain Applications in Healthcare – A Review and Future Perspective
Natsiavas et al. (2019)	Citizen Perspectives on Cross-Border eHealth Data Exchange: A European Survey
Neumann et al. (2019)	Strategies to digitalize inert health practices: The gamification of glucose monitoring
Nokkala and Dahlberg (2019)	Empowering citizens through data interoperability - data federation applied to consumer-centric healthcare
Omarov et al. (2021)	Internet of Things in Healthcare: A Review
Oshni Alvandi et al. (2021)	Understanding digital health ecosystem from Australian citizens' perspective: A scoping review
Pandey et al. (2021)	Security of Healthcare Data Using Blockchains: A Survey
Paul et al. (2021)	Industry 4.0 Applications for Medical/Healthcare Services
Philip et al. (2020)	Medchain-Medical Record Securance Using Blockchain
Rastogi et al. (2022)	Risk Stratification for Subjects Suffering from Lung Carcinoma: Healthcare 4.0 Approach with Medical Diagnosis Using Computational Intelligence
Ricciardi et al. (2019)	How to govern the digital transformation of health services
Schulz and Schönheit (2021)	Value-Added Process Design for Digital Transformation in Hospitals and Medical Networks

-continued-

Author (year)	Title
Spena and Cristina (2019)	Practising innovation in the healthcare ecosystem: the agency of third-party actors
Sulaiman et al. (2021)	AI-Enabled Proactive mHealth: A Review
Veeraiah and Ravikumar (2020)	Integrated Health Care Delivery system with IoT Enabling Technology
Vogt et al. (2019)	Digital Transformation in Healthcare: How the Potential of Digital Health Is Tackled to Transform the Care Process of Intensive Care Patients Across All Healthcare Sectors
Warraich et al. (2018)	The digital transformation of medicine can revitalize the patient-clinician relationship
Wojturska (2021)	Medical data security in digital transformation of healthcare: Assessment of e-Health solutions standarisation in the European Union
Wong et al. (2022)	The dawn of digital public health in Europe: Implications for public health policy and practice
Yang et al. (2021)	Multimodal Wearable Intelligence for Dementia Care in Healthcare 4.0: a Survey
Ziadlou (2021)	Strategies during digital transformation to make progress in achievement of sustainable development by 2030
Zippel-Schultz et al. (2017)	Current status and future of telemonitoring: Scenarios for telemedical care in 2025
